# Severity stratification of NICU-admitted neonates using Robson classification and obstetric risk profile: a nomogram-based study

**DOI:** 10.3389/fmed.2026.1812229

**Published:** 2026-07-08

**Authors:** Seniye Burcu Torumtay Alic, Gulcin Aydogdu

**Affiliations:** 1 Department of Gynecology and Obstetrics, Faculty of Medicine, Hitit University, Çorum, Türkiye; 2 Department of Biostatistics, Faculty of Medicine, Hitit University, Çorum, Türkiye

**Keywords:** gestational age, level of neonatal care, mode of delivery, neonatal intensive care unit, obstetric risk factors, nomogram, Robson classification

## Abstract

**Background:**

Admission to a neonatal intensive care unit (NICU) reflects neonatal morbidity and the downstream impact of obstetric decision-making. While most previous studies have focused on NICU admission as a binary outcome, the determinants of the required level of care remain poorly understood. This study aimed to evaluate whether the Robson classification, which categorises pregnancies according to obstetric characteristics such as parity, fetal presentation, gestational age, prior cesarean section, and onset of labor, together with obstetric risk profile, can stratify the severity of care among neonates admitted to the NICU and identify factors associated with level 3 NICU care.

**Methods:**

This retrospective, single-center study included 1,815 neonates admitted to the NICU between January 2023 and December 2025 among 7,632 total deliveries. Maternal, obstetric, and neonatal characteristics were analyzed across NICU levels (1–3). Univariable and multivariable binary logistic regression analyses were performed to identify independent predictors of level 3 NICU care (vs. levels 1–2). A nomogram was constructed to estimate individualized risk. Model performance was evaluated using discrimination (C-index), calibration (calibration plot and Hosmer–Lemeshow test), and internal validation via bootstrap resampling (1,000 iterations).

**Results:**

Overall, 40.4% of NICU-admitted neonates required level 3 care. Higher NICU levels were associated with lower gestational age and birth weight (*p* < 0.001). Each additional week of gestation reduced the likelihood of level 3 care (OR = 0.82, 95% CI: 0.79–0.85). A history of miscarriage was associated with lower odds of level 3 care (OR = 0.15, 95% CI: 0.08–0.28), although this finding should be interpreted with caution. Neonates born to mothers classified as non–low risk according to the Robson classification had higher odds of level 3 care (OR = 1.46, 95% CI: 1.18–1.79). The model demonstrated modest discrimination (C-index: 0.695; optimism-corrected: 0.694), good calibration, and consistent performance across clinically relevant threshold probabilities.

**Conclusion:**

Gestational age, obstetric risk profile, and Robson classification contribute to stratifying the severity of care among NICU-admitted neonates. The proposed model demonstrated modest discriminatory performance and should be interpreted primarily as an exploratory risk-stratification framework rather than a clinically actionable prediction tool. Although it may help contextualize obstetric risk patterns among NICU-admitted neonates, external validation and refinement are required before any broader clinical application.

## 1 Introduction

Admission to the Neonatal Intensive Care Unit (NICU) is an important indicator of neonatal morbidity and mortality and reflects the short- and long-term outcomes of obstetric practices. The need for NICU admission depends on multiple variables, including maternal characteristics, pregnancy-related factors, mode of delivery, and fetal condition. NICU care is commonly categorized into levels according to the intensity of clinical support required, ranging from basic care (level 1) to advanced intensive care (level 3), which includes premature, critically ill, and complex cases. Therefore, identifying factors associated not only with NICU admission but also with the level of care required is of considerable clinical importance (1, 2).

Although factors such as maternal age, parity, multiple pregnancy, adverse obstetric history, and gestational age are known to be associated with NICU admission, the determinants of care intensity among NICU-admitted neonates have been less extensively studied (2, 3). In particular, defining clinical markers associated with higher-level NICU care may contribute to exploratory risk stratification and improved understanding of factors associated with neonatal care intensity (4).

The impact of mode of delivery on neonatal outcomes has long been debated in the literature. Evidence suggests that cesarean deliveries performed for elective or fetal indications may increase NICU admission rates (5, 6). However, the specific conditions under which cesarean delivery increases or reduces neonatal risks remain unclear and have not been sufficiently addressed in detail (7). In recent years, the Robson classification has been used as a standardized tool for evaluating deliveries based on obstetric characteristics, enabling systematic comparisons of delivery practices and associated outcomes. Nevertheless, studies investigating the association between Robson groups and NICU care levels remain limited (7, 8). Furthermore, analyzing cesarean section indications by categorizing them into maternal, fetal, and placental causes and relating them to the severity of NICU care may provide additional clinically relevant insights.

In this study, we evaluated the relationship between NICU admission levels and Robson classification, mode of delivery, cesarean section indications, maternal demographic characteristics, obstetric history, gestational age, and pregnancy characteristics among neonates admitted to the NICU. In addition, we aimed to identify factors associated with level 3 NICU care, representing the most severe clinical condition, and to develop an exploratory nomogram-based framework for severity stratification within this population. Our study seeks to provide exploratory insights into the obstetric factors associated with NICU care intensity.

## 2 Materials and methods

### 2.1 Study design and population

This study was designed as a retrospective observational cohort study evaluating neonates admitted to the neonatal intensive care unit (NICU). A total of 1,815 neonates admitted to the NICU among 7,632 deliveries that occurred at Hitit University Erol Olçok Training and Research Hospital between January 1, 2023, and December 31, 2025, were included in the study.

The study population was restricted to NICU-admitted neonates; therefore, the analytical framework was designed to assess severity of care within this cohort, rather than predicting NICU admission in the general obstetric population.

Mode of delivery was recorded as vaginal delivery or cesarean section.

### 2.2 Data collection and variables

Study data were obtained from the hospital electronic medical record system, delivery room records, and NICU databases. For each case, maternal demographic characteristics, obstetric history, pregnancy- and delivery-related information, and neonatal data were recorded using a standardized data collection form.

Maternal variables included maternal age, nationality (Republic of Türkiye citizen / other), gravidity, number of live births, history of stillbirth, and history of miscarriage. Obstetric variables included mode of delivery, primiparity, operative vaginal delivery, multiple pregnancy status, and gestational age.

For obstetric classification, all cases were grouped according to the Robson classification system. Robson groups 1 and 3 were defined as the low-risk group, while the remaining groups (2, 4–10) were categorized as non–low-risk. Although the Robson classification was originally developed to monitor cesarean section rates rather than to define neonatal risk, groups 1 and 3 generally represent singleton, term, cephalic pregnancies with spontaneous labor and without previous cesarean delivery, which are commonly considered lower obstetric complexity populations in clinical practice. Therefore, for exploratory risk stratification purposes, these groups were categorized separately from the remaining Robson groups.

This simplification was intentionally adopted to improve model stability and interpretability within the available sample size; however, it inevitably reduces the clinical granularity of the original Robson system and may obscure important heterogeneity between individual obstetric subgroups. Therefore, the resulting classification should be interpreted as a pragmatic exploratory grouping rather than a biologically or clinically homogeneous risk construct.

Among cesarean deliveries, indications were classified into maternal, fetal, and placental categories. In cases with multiple indications, the primary clinical indication was recorded.

Neonatal variables included sex, birth weight (g), birth length (cm), and duration of NICU stay. Gestational age was analyzed both as a continuous variable and categorically (preterm <37 weeks; term ≥37 weeks).

### 2.3 Outcome definition and prediction framework

The primary outcome of the study was NICU care level, classified as level 1, level 2, or level 3 based on the intensity of clinical support required. For prediction modeling, level 3 NICU care (representing the most severe clinical condition) was analyzed as a binary outcome against levels 1–2.

The decision to combine levels 1 and 2 was based on both clinical and statistical considerations. Clinically, these groups represent neonates not requiring invasive intensive care, whereas level 3 reflects advanced critical care. Statistically, the relatively small proportion of level 1 cases (15.0%) limited the stability of an ordinal model. Nevertheless, this approach may have reduced discrimination between intermediate and advanced neonatal care profiles.

The intended prediction timepoint of the model was the late antenatal or immediate pre-delivery period, using variables available before birth. Postnatal variables such as birth weight, neonatal length, and NICU length of stay were included only for descriptive and exploratory analyses and were not considered eligible predictors in the final multivariable model.

NICU admission levels were defined based on the intensity of care required at admission, in accordance with international guidelines (American Academy of Pediatrics, British Association of Perinatal Medicine, and World Health Organization) (9, 11, 12), and adapted to local clinical practice. To enhance reproducibility and ensure clarity across different clinical settings, the operational definitions used in this study are presented in Table 1.

**Table 1:** Operational definitions of NICU care levels.

NICU level	Clinical description	Key interventions/criteria
Level 1 (basic care)	Stable neonates requiring minimal monitoring	Routine care, no respiratory support or only short-term oxygen, oral feeding, basic monitoring
Level 2 (intermediate care)	Moderately ill neonates requiring non-invasive support	Non-invasive respiratory support (e.g., CPAP), intravenous fluids, partial parenteral nutrition, continuous monitoring
Level 3 (intensive care)	Critically ill or premature neonates requiring advanced support	Mechanical ventilation, invasive respiratory support, vasoactive drugs, total parenteral nutrition, invasive monitoring, management of multi-organ dysfunction

NICU level was recorded according to the highest level of care required at admission.

### 2.4 Statistical analysis

Statistical analyses were conducted using IBM SPSS Statistics (Version 22.0) and Python. Continuous variables were assessed for normality using the Kolmogorov–Smirnov test and graphical methods. Non-normally distributed variables were summarized as median (minimum–maximum), while categorical variables were presented as frequency and percentage.

Comparisons across NICU levels were performed using the Kruskal–Wallis test for continuous variables and chi-square or Fisher’s exact test for categorical variables. *Post hoc* analyses were conducted using Dunn–Bonferroni correction.

Before multivariable modeling, correlations among continuous predictors were evaluated using Spearman correlation to assess potential collinearity.

Univariable and multivariable binary logistic regression analyses were performed to identify predictors of level 3 NICU care. Candidate variables were selected based on a hybrid approach combining (1) clinical relevance supported by the literature and (2) statistical significance in univariable analysis (*p* < 0.10). Variables were not selected solely on statistical grounds. Given the moderate number of candidate predictors and the clinically hypothesis-driven nature of the analysis, a conventional multivariable logistic regression framework was preferred. However, future studies using penalized regression approaches may provide more stable variable selection.

Model performance was evaluated using discrimination (C-index), calibration (Hosmer–Lemeshow test and calibration plot), and internal validation via bootstrap resampling (1,000 iterations).

A nomogram was constructed based on the final multivariable model to estimate individualized risk.

As a sensitivity analysis, an ordinal logistic regression model including NICU levels (1–3) was performed to confirm the robustness of the findings.

All statistical tests were two-sided, and *p* < 0.05 was considered statistically significant.

### 2.5 Missing data and model assumptions

A systematic assessment of missing data was performed for all variables included in the regression analyses. No missing data were identified (0/1,815), and therefore no imputation was required. The absence of missing data reflects the use of a mandatory structured electronic obstetric and neonatal database routinely completed at delivery and NICU admission within the institution.

The linearity assumption for continuous variables was evaluated using the Box–Tidwell procedure. The linearity of gestational age in the logit was confirmed (*p* = 0.56).

### 2.6 Reporting standards

This study was conducted and reported in accordance with the TRIPOD+AI guidelines for multivariable prediction models. The completed TRIPOD+AI checklist is provided as Supplementary material (Supplementary File 1).

## 3 Results

A total of 1,815 neonates were included in the study. Of these, 15.0% (*n* = 273) were admitted to NICU level 1, 44.5% (*n* = 808) to level 2, and 40.4% (*n* = 734) to level 3 (Table 2).

**Table 2:** Comparison of demographic and obstetric characteristics according to NICU level.

Variables	NICU level 1	NICU level 2	NICU level 3	*p*-value
Maternal age (years)	28 (18–46)	30 (16–48)	29 (17–56)	0.185^a^
Nationality				
Turkish citizen	252 (15.1%)	741 (44.3%)	681 (40.7%)	0.734^b^
Other national	21 (14.9%)	67 (47.5%)	53 (37.6%)	
Parity				
1	268 (16.2%)	748 (45.2%)	639 (38.6%)	<0.001^b^
≥2	5 (3.1%)	60 (37.5%)	95 (59.4%)	
History of miscarriage				
No	245 (14.3%)	746 (43.5%)	723 (42.2%)	<0.001^b^
Yes	28 (27.7%)	62 (61.4%)	11 (10.9%)	
History of stillbirth				
No	272 (15%)	807 (44.6%)	730 (40.4%)	0.320^c^
Yes	1 (16.7%)	1 (16.7%)	4 (66.7%)	
Total	273 (15%)	808 (44.5%)	734 (40.4%)	

^a^Kruskal–Wallis test (median, min–max), ^b^Chi-Square test, *n* (%)–row percentages, ^c^Fisher’s Exact test, *n* (%)–row percentages.

No statistically significant differences were observed among NICU admission levels with respect to maternal age and maternal nationality (*p* = 0.185 and *p* = 0.734, respectively). In contrast, significant differences were found in parity and history of miscarriage (*p* < 0.001). Neonates born to mothers with parity ≥2 had a higher proportion of level 3 NICU care compared with those with ≤1 live birth (*p* < 0.001).

In descriptive analyses, neonates born to mothers with a history of miscarriage were more frequently admitted to level 2 NICU care; however, this variable demonstrated a different association in multivariable analysis. No statistically significant difference was observed in relation to a history of stillbirth (*p* = 0.320; Table 2).

When pregnancy- and delivery-related characteristics were evaluated, gestational age, mode of delivery, and type of pregnancy differed significantly across NICU levels (*p* < 0.001; Table 3). Gestational age decreased progressively with increasing NICU level (all *post hoc* comparisons *p* < 0.001).

**Table 3:** Pregnancy and delivery related characteristics by NICU level.

Variables	NICU level 1	NICU level 2	NICU level 3	*p*-value	*Post hoc* *p*-value
Gestational age (weeks)	39 (25–43)	38 (24–43)	36 (20–41)	<0.001^a^	1–2: <0.001 1–3: <0.001 2–3: <0.001
Mode of delivery					
Vaginal delivery	122 (19.1%)	302 (47.3%)	214 (33.5%)	<0.001^b^	
Cesarean section	151 (12.8%)	506 (43.0%)	520 (44.2%)		
Type of pregnancy					
Singleton pregnancy	268 (16.2%)	751 (45.3%)	639 (38.5%)	<0.001^b^	
Multiple pregnancy	5 (3.2%)	57 (36.3%)	95 (60.5%)		
Gestational age category					
Preterm	39 (5.4%)	292 (40.3%)	393 (54.3%)	<0.001^b^	
Term	234 (21.4%)	516 (47.3%)	341 (31.3%)		
Robson risk group					
Low-risk	199 (17.2%)	542 (47.0%)	413 (35.8%)	<0.001^b^	
Non–low-risk	74 (11.2%)	266 (40.2%)	321 (48.6%)		
Total	273 (15%)	808 (44.5%)	734 (40.4%)		

Non–low-risk: Robson 2, 4, 5, 6, 7, 8, 9, 10 and Low-risk: Robson 1, 3. ^a^Kruskal–Wallis test (median, min–max). ^b^Chi-Square test, *n* (%)–row percentages. ^c^Fisher’s Exact test, *n* (%)–row percentages.

The proportion of level 3 NICU care was higher among neonates delivered by cesarean section compared with vaginal delivery (44.2% vs. 33.5%). Multiple pregnancies and preterm birth were also associated with a higher proportion of level 3 NICU care (*p* < 0.001). In addition, neonates in the non–low-risk Robson group had a higher proportion of level 3 NICU care than those in the low-risk group (*p* < 0.001; Table 3).

With respect to neonatal characteristics, birth weight and birth length differed significantly across NICU levels (*p* < 0.001; Table 4). Neonates admitted to level 3 had significantly lower birth weight and shorter birth length compared with those in levels 1 and 2 (*p* < 0.001).

**Table 4:** Neonatal characteristics according to NICU level.

Variables	NICU level 1	NICU level 2	NICU level 3	*p*-value	*Post hoc* *p*-value
Birth weight (grams)	3,240 (620–4,640)	2,975 (490–4,600)	2,635 (310–5,290)	<0.001^a^	1-2: <0.001 1–3: <0.001 2–3: <0.001
Birth length (centimeters)	50 (30–54)	50 (27–55)	48 (24–56)	<0.001^a^	1-2: <0.001 1–3: <0.001 2–3: <0.001
Length of NICU stay (days)	2 (1–7)	2 (1–26)	2 (1–24)	<0.001^a^	1-2: 0.020 1–3: <0.001 2–3: <0.001
Neonatal sex					
Female	120 (15.8%)	331 (43.6%)	309 (40.7%)	0.679^b^	
Male	153 (14.5%)	477 (45.2%)	425 (40.3%)		
Birth weight category					
Extremely low birth weight	2 (4.9%)	4 (9.8%)	35 (85.4%)	<0.001^b^	
Very low birth weight	0 (0%)	13 (25%)	39 (75%)		
Low birth weight	24 (5.2%)	194 (42.3%)	241 (52.5%)		
Normal birth weight	228 (19.4%)	555 (47.3%)	391 (33.3%)		
Macrosomia	18 (24.0%)	37 (49.3%)	20 (26.7%)		
Severe macrosomia	1 (7.1%)	5 (35.7%)	8 (57.1%)		
Total	273 (15%)	808 (44.5%)	734 (40.4%)		

^a^Kruskal–Wallis test (median, min–max), ^b^Chi-square test, *n* (%)–row percentages.

Length of NICU stay also differed significantly across levels (*p* < 0.001), with wider variability and generally longer stays observed in higher levels of care.

No statistically significant difference was observed in neonatal sex distribution (*p* = 0.679). However, extremely low birth weight and very low birth weight neonates were predominantly admitted to level 3 NICU care (*p* < 0.001; Table 4).

In subgroup analyses, no significant differences were observed in parity or operative vaginal delivery within the vaginal delivery group (*p* = 0.514 and *p* = 0.067, respectively). However, preterm vaginal delivery was associated with a higher proportion of level 3 NICU care (*p* < 0.001).

Within the cesarean section subgroup, no significant differences were found between primary and repeat cesarean deliveries (*p* = 0.269). However, cesarean deliveries in multiple pregnancies and preterm cesarean deliveries were associated with higher rates of level 3 NICU care (*p* < 0.001; Table 5). No significant association was observed between cesarean indication categories and NICU level (*p* = 0.585).

**Table 5:** Vaginal and cesarean delivery subgroup analyses according to NICU admission level.

Variables	NICU level 1	NICU level 2	NICU level 3	*p*-value
Vaginal delivery subgroup analyses				
Non-first	50 (17.2%)	142 (48.8%)	99 (34%)	0.514^a^
First	72 (20.7%)	160 (46.1%)	115 (33.1%)	
Non-instrumental	119 (19.2%)	297 (48%)	203 (32.8%)	0.067^a^
Instrumental	3 (15.8%)	5 (26.3%)	11 (57.9%)	
Preterm	10 (6.4%)	66 (42.0%)	81 (51.6%)	<0.001^a^
Term	112 (23.3%)	236 (49.1%)	133 (27.7%)	
Total	122 (19.1%)	302 (47.3%)	214 (33.5%)	
Cesarean section subgroup analyses				
Primary	82 (14%)	256 (43.8%)	246 (42.1%)	0.269^a^
Repeat	69 (11.6%)	250 (42.2%)	274 (46.2%)	
Singleton	146 (14.3%)	449 (43.8%)	429 (41.9%)	<0.001^a^
Multiple pregnancy	5 (3.3%)	57 (37.3%)	91 (59.5%)	
Cesarean section indication category				
Maternal	83 (13.2%)	275 (43.9%)	269 (42.9%)	0.585^b^
Fetal	66 (12.2%)	225 (41.7%)	248 (46%)	
Placental	2 (18.2%)	6 (54.5%)	3 (27.3%)	
Preterm	29 (5.1%)	226 (39.9%)	312 (55.0%)	<0.001^a^
Term	122 (20.0%)	280 (45.9%)	208 (34.1%)	
Total	151 (12.8%)	506 (43%)	520 (44.2%)	

^a^Chi-Square test, *n* (%)–row percentages, ^b^Fisher’s Exact test, *n* (%)–row percentages.

Univariable and multivariable logistic regression analyses were performed to identify factors associated with level 3 NICU care (Table 6). Birth weight, neonatal length, and length of hospital stay were excluded from the predictive model as they represent postnatal variables.

**Table 6:** Results of univariable and multivariable binary logistic regression analyses performed to identify factors associated with level 3 NICU admission.

Variables	Univariable		Multivariable	
	*p*-value	OR (95% CI)	*p*-value	OR (95% CI)
Nationality	0.473	1.14 (0.80–1.62)	ns	–
Gestational age (weeks)	<0.001	0.81 (0.78–0.84)	<0.001	0.82 (0.79–0.85)
Mode of delivery (Cesarean vs. Vaginal)	<0.001	1.57 (1.28–1.92)	ns	–
Multiple pregnancy (yes vs. no)	<0.001	2.44 (1.75–3.42)	ns	–
Maternal age (years)	0.331	1.01 (0.99–1.02)	ns	–
Parity (≥2 vs. 1)	<0.001	2.32 (1.67–3.23)	ns	–
History of stillbirth (yes vs. no)	0.211	2.96 (0.54–16.18)	ns	–
History of miscarriage (yes vs. no)	<0.001	0.17 (0.09–0.32)	<0.001	0.15 (0.08–0.28)
Robson risk group (non-low vs. low)	<0.001	1.69 (1.40–2.06)	<0.001	1.46 (1.18–1.79)
Neonatal sex (male vs. female)	0.873	0.99 (0.81–1.19)	ns	–

Multivariable model: Nagelkerke *R*^2^ = 0.160, Overall classification accuracy = 66.2%, ns, not significant; OR, odds ratio; CI, confidence interval.

In the multivariable model, gestational age, history of miscarriage, and Robson classification were identified as independent factors associated with level 3 NICU care. Each additional week of gestation was associated with a reduced likelihood of level 3 care (OR = 0.82; 95% CI: 0.79–0.85; *p* < 0.001). A history of miscarriage was associated with a lower likelihood of level 3 NICU care (OR = 0.15; 95% CI: 0.08–0.28; *p* < 0.001). Neonates in the non–low-risk Robson group had a higher likelihood of level 3 NICU care compared with the low-risk group (OR = 1.46; 95% CI: 1.18–1.79; *p* < 0.001).

The model demonstrated only modest discriminatory ability, with an apparent C-index of 0.695 and an optimism-corrected C-index of 0.694, indicating limited capacity for individualized clinical prediction despite acceptable calibration. Calibration was acceptable, as indicated by the Hosmer–Lemeshow test (*p* = 0.328). The Brier score was 0.212, indicating reasonable overall model performance.

At a threshold probability of 0.40, the model achieved a sensitivity of 0.67 and specificity of 0.61. At a lower threshold of 0.30, sensitivity increased to 0.83 with reduced specificity (0.40), reflecting the expected trade-off between sensitivity and specificity. Performance metrics across clinically relevant threshold probabilities are presented in Supplementary Table S1. These threshold analyses are presented primarily to illustrate the operating characteristics of the model across different probability cutoffs rather than to define clinically validated intervention thresholds.

**Figure 1:**
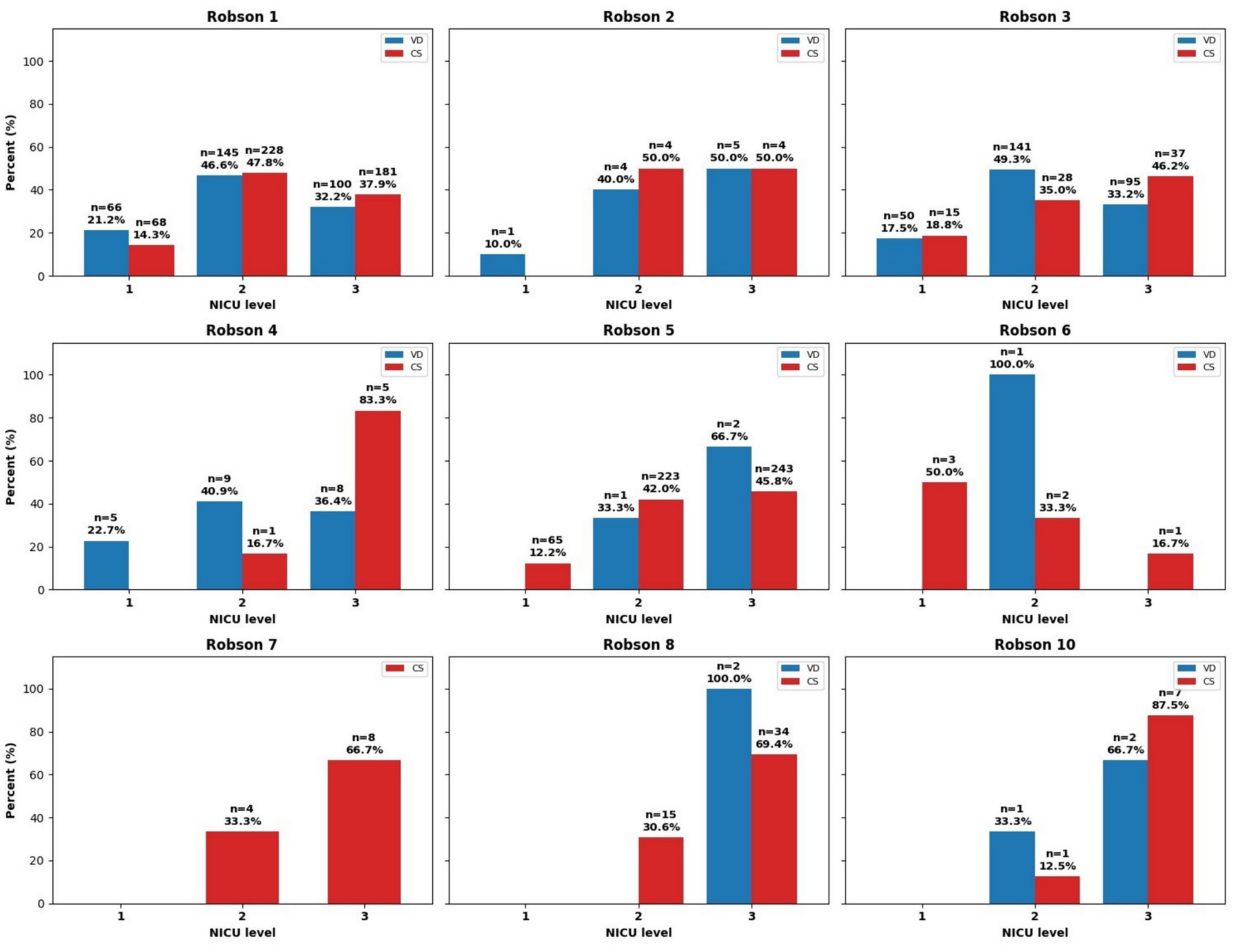
Distribution of NICU admission levels according to mode of delivery across Robson groups Some Robson groups contained very small sample sizes, and results in these subgroups should be interpreted with caution.

**Figure 2:**
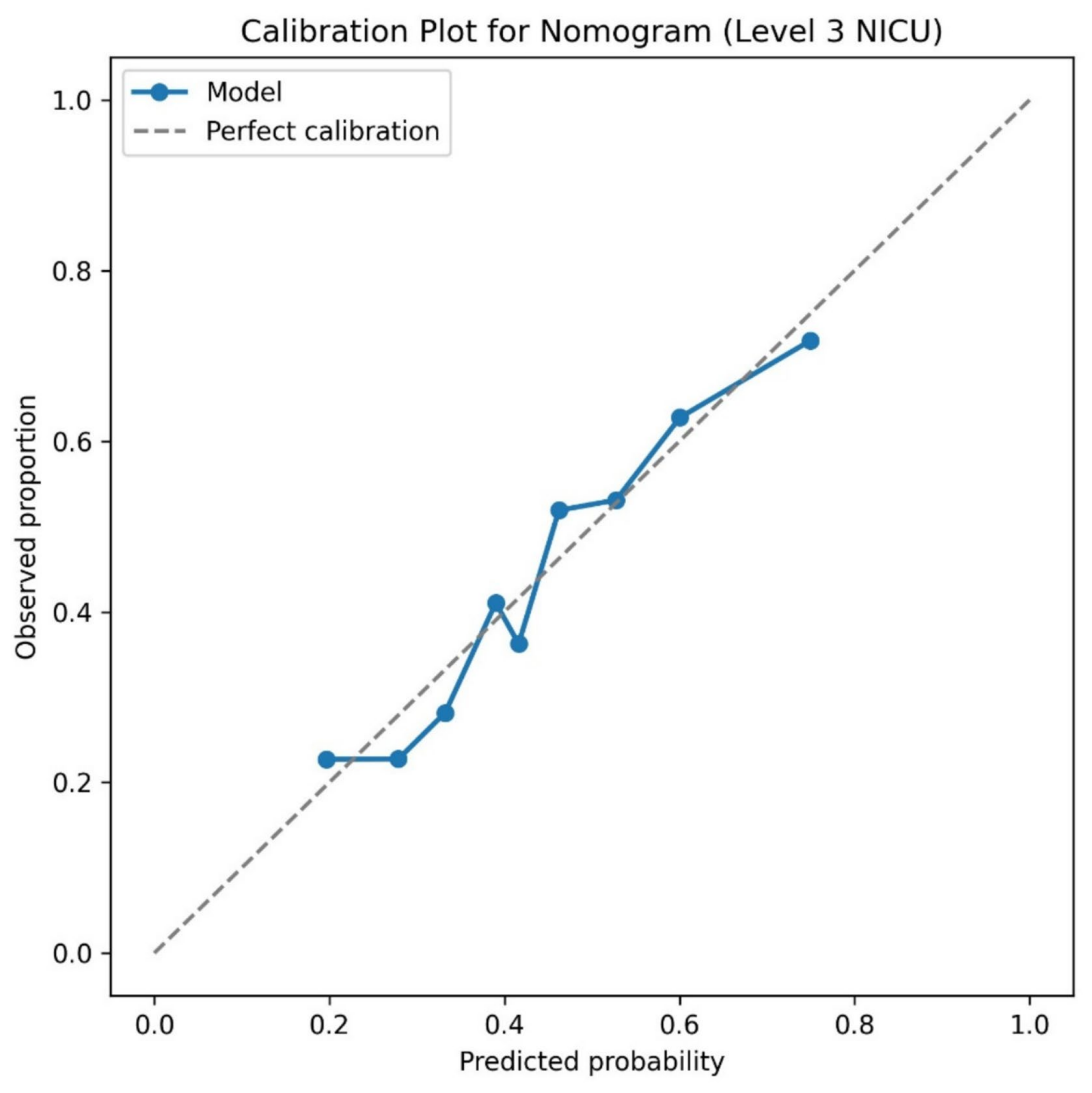
Calibration plot of the model for level 3 NICU care. The dashed diagonal line represents perfect calibration, while the solid line indicates the model’s performance. The close alignment between the two lines demonstrates good agreement between predicted and observed probabilities.

Figure 1 illustrates the distribution of NICU admission levels according to mode of delivery within each Robson group. A nomogram based on the final multivariable model is presented in Supplementary material, and the calibration plot is shown in Figure 2.

## 4 Discussion

In this study, we evaluated factors associated with NICU care intensity among NICU-admitted neonates and identified variables independently associated with level 3 NICU care. The main findings indicate that lower gestational age, non–low-risk Robson classification, and the absence of miscarriage history were associated with a higher likelihood of level 3 care. These results suggest that, within a cohort already admitted to the NICU, variation in care intensity appears to be associated with underlying obstetric risk characteristics.

Gestational age emerged as the most robust and consistent factor associated with level 3 NICU care. Each additional week of gestation was associated with an approximately 18% reduction in the likelihood of level 3 care. This finding is consistent with previous literature demonstrating that prematurity is strongly associated with increased need for respiratory support, invasive procedures, and advanced monitoring. In this context, gestational age appears to represent a fundamental clinical determinant of care intensity among NICU-admitted neonates rather than merely a correlate of admission status (2, 4).

The finding related to miscarriage history requires particularly cautious interpretation. In descriptive analyses, neonates born to mothers with a history of miscarriage were more frequently admitted to level 2 NICU care, whereas multivariable analysis showed a markedly lower likelihood of level 3 care. Given the magnitude of this association, it should not be interpreted as evidence of a causal protective effect. More plausibly, it may reflect residual confounding, selection bias, differences in antenatal surveillance, or documentation-related factors. It is also possible that pregnancies progressing to viability after prior miscarriage represent a selected subgroup with distinct clinical characteristics. Further studies with more detailed obstetric-history data are required to clarify this association. In addition, a sensitivity analysis excluding miscarriage history from the multivariable model yielded materially similar results, supporting the stability of the main associations observed.

Although cesarean delivery was associated with a higher proportion of level 3 NICU care in descriptive analyses, this relationship likely reflects underlying obstetric conditions such as prematurity, multiple pregnancy, and fetal compromise rather than an independent effect of delivery mode (5–7, 13, 14).

A notable finding of this study was the association between non–low-risk Robson grouping and a higher likelihood of level 3 NICU care. Although the Robson classification was originally developed to standardize cesarean section monitoring, our findings suggest that, within this selected NICU cohort, it may capture clinically relevant aspects of the obstetric context associated with neonatal care intensity. However, this result should be interpreted cautiously. Collapsing Robson groups into low-risk and non–low-risk categories reduces clinical granularity and combines heterogeneous obstetric scenarios. As a result, the observed association may partly reflect broad differences in obstetric complexity rather than a distinct contribution of the simplified Robson grouping itself. Therefore, the present findings support an association rather than demonstrating clear incremental predictive value. Future studies using full-category Robson modeling or alternative hierarchical approaches may better preserve clinical heterogeneity and clarify whether the observed signal remains independent of core obstetric variables (8).

Birth weight, birth length, and NICU length of stay differed significantly across NICU levels. However, these variables represent postnatal measurements and should be interpreted as descriptive correlates of care intensity rather than predictors within the intended modeling framework. Their associations reinforce the internal clinical gradient across NICU levels but do not define the predictive structure of the model. Previous studies have likewise reported strong relationships between prematurity, low birth weight, prolonged hospitalization, and higher neonatal care requirements (4, 14–16).

The multivariable model demonstrated modest discrimination, with an apparent C-index of 0.695 and an optimism-corrected C-index of 0.694. Although calibration was acceptable and internal validation suggested limited overfitting, the discriminatory performance remains insufficient for individualized clinical prediction or stand-alone decision support. Accordingly, the nomogram should be interpreted as an exploratory analytical representation of associations observed within this selected NICU cohort rather than as a clinically actionable prediction instrument. Its potential value may lie primarily in hypothesis generation and structured risk contextualization pending external validation and refinement in broader obstetric populations.

Several limitations should be considered. First, the study included only neonates admitted to the NICU; therefore, the model does not predict NICU admission or level 3 care in the overall obstetric population but rather stratifies severity within an already selected cohort. Second, the retrospective single-center design may limit generalizability and reflect institution-specific admission and classification practices. Third, although NICU levels were operationally defined, classification may still be influenced by local clinical decision-making. Fourth, the simplified dichotomization of Robson groups substantially reduces the clinical granularity of the original classification system and may obscure heterogeneous associations between individual obstetric subgroups and NICU care intensity, potentially limiting both interpretability and predictive performance. Fifth, although a clinically informed variable selection strategy was used, more advanced modeling approaches such as penalized regression could provide more robust predictor selection. The observed C-index values suggest only moderate separation between outcome groups, reinforcing that the present model is more suitable for exploratory stratification than for high-confidence clinical prediction. Finally, the strong association observed for miscarriage history may reflect residual confounding or selection bias and requires further investigation.

Despite these limitations, the study has several strengths. It addresses a clinically relevant but underexplored question, includes a relatively large NICU cohort, applies multivariable modeling with internal validation, and shifts the analytical focus from binary NICU admission toward variation in care intensity among NICU-admitted neonates.

## 5 Conclusion

Among NICU-admitted neonates, gestational age, miscarriage history, and Robson risk grouping were associated with level 3 NICU care. Gestational age was the most consistent determinant, reinforcing the central role of prematurity in neonatal care intensity (2, 4). The proposed model demonstrated modest discriminatory performance and should be interpreted as an exploratory severity-stratification framework within a selected NICU population rather than a clinically actionable prediction model. More granular obstetric modeling, external validation, and evaluation across broader populations are required before potential clinical utility can be meaningfully assessed.

## Data Availability

The raw data supporting the conclusions of this article will be made available by the authors, without undue reservation.

## Supplementary material

SUPPLEMENTARY FIGURE S1 Nomogram for predicting the probability of level 3 NICU admission. Nomogram developed based on the final multivariable logistic regression model to estimate the individualized probability of level 3 NICU admission. The model incorporates gestational age, Robson risk group (low-risk vs non–low-risk), and history of miscarriage (yes/no). Each predictor is assigned a corresponding point value, and the total score corresponds to the predicted probability of level 3 NICU admission.
