# Supplementary material for: Severity stratification of NICU-admitted neonates using Robson classification and obstetric risk profile: a nomogram-based study
Source: Front Med (Lausanne). 2026 Jul 8;13:1812229. doi: 10.3389/fmed.2026.1812229 (PMC13388305; doi:10.3389/fmed.2026.1812229)
Supplement: Supplementary file 1 [file Table_1.DOCX]

**Supplementary Material**

*Prediction of Level 3 Neonatal Intensive Care Unit Admission Using Robson Classification and Obstetric Risk Profile: A Nomogram-Based Study*

**TRIPOD+AI Reporting Checklist**

*Collins GS, et al. TRIPOD+AI statement: updated guidance for reporting clinical prediction models that use regression or machine learning methods. BMJ. 2024;385:e078378.*

| **Section** | **Item** | **Checklist Item** | **Reported?** | **Location** | **Comment** |
| --- | --- | --- | --- | --- | --- |
| Title & Abstract | **1** | Identify the study as developing and/or validating a multivariable prediction model, the target population, and the outcome to be predicted. | **Yes** | Title Page | Title explicitly states nomogram-based study for Level 3 NICU admission prediction. |
| Abstract | **2** | Provide a structured summary including study design, setting, participants, sample size, predictors, outcome, statistical methods, results, and conclusions. | **Yes** | Abstract | Structured abstract covers all required elements including C-index, calibration, and DCA. |
| Introduction | **3a** | Explain the medical context and rationale for developing or validating the multivariable prediction model. | **Yes** | Section 1 | Introduction details clinical burden of NICU admission and rationale for level-based prediction. |
| Introduction | **3b** | Specify the objectives, including whether the study describes development, validation, or updating of a model. | **Yes** | Section 1 | Study objective explicitly stated: development of a nomogram for Level 3 NICU admission. |
| Methods — Data | **4a** | Describe the study design and setting, including start and stop dates of accrual. | **Yes** | Section 2 | Retrospective cohort; Jan 2023–Dec 2025; Hitit University Erol Olcok Training and Research Hospital, Turkey. |
| Methods — Data | **4b** | Describe the eligibility criteria for participants. | **Yes** | Section 2 | All neonates admitted to NICU among 7,632 deliveries; N=1,815 included. |
| Methods — Data | **5a** | Describe the outcome(s) to be predicted, including how and when assessed. | **Yes** | Section 2 | Primary outcome: Level 3 NICU admission (binary: Level 3 vs. Levels 1+2). Defined by clinical care requirements at admission. |
| Methods — Data | **5b** | Report any actions to blind assessment of predictors for the outcome. | **Yes** | Section 2 | Retrospective data extraction from electronic records. |
| Methods — Data | **6a** | Describe the predictors used, including how and when they were measured. | **Yes** | Section 2 | 13 candidate predictors described; all measured at or before delivery. Three predictors retained in final model. |
| Methods — Data | **7** | Explain how the study size was arrived at. | **Yes** | Section 2 | All eligible neonates within study period included (N=1,815; events=734). EPV ratio ~245 for final model. |
| Methods — Data | **8** | Describe how missing data were handled. | **Yes** | Section 2.1 (revised) | Complete data on all candidate predictors (0/1,815 missing). No imputation required. |
| Methods — Model | **9** | Describe how predictors were handled in the analyses. | **Yes** | Section 2.1 | Gestational age: continuous, linearity confirmed (Box-Tidwell p=0.56). Miscarriage history and Robson group: binary. |
| Methods — Model | **10a** | Specify the type of model, all model-building procedures, and method for internal validation. | **Yes** | Section 2.1 | Binary logistic regression; univariable p<0.10 screening then multivariable; bootstrap internal validation (1,000 iterations). |
| Methods — Model | **10b** | For validation, describe the method for assessing model performance. | **Yes** | Section 2.1 | C-index, calibration plot, Hosmer-Lemeshow test, bootstrap optimism correction, Brier Score, DCA. |
| Methods — Model | **10c** | Describe any model updating arising from the validation. | **N/A** | N/A | Development study only; no external validation performed. |
| Methods — Model | **11** | Provide details on any interface developed. | **N/A** | N/A | Nomogram constructed; no digital interface implemented. |
| Results | **12** | Describe flow of participants, including number with and without the outcome. | **Yes** | Section 3 / Table 1 | N=1,815: Level 1: 273 (15.0%); Level 2: 808 (44.5%); Level 3: 734 (40.4%). |
| Results | **13a** | Report characteristics of participants, including number with missing data. | **Yes** | Tables 1-3 | All demographic, obstetric, and neonatal characteristics reported. No missing data on predictors. |
| Results | **13b** | For validation, report characteristics of validation data. | **N/A** | N/A | Internal validation only (bootstrap). |
| Results | **14a** | Specify the number of participants and outcome events in each analysis. | **Yes** | Section 3 / Table 5 | N=1,815; Level 3 events=734 (40.4%); complete case analysis. |
| Results | **14b** | Report the unadjusted association between each candidate predictor and outcome. | **Yes** | Table 5 | Univariable ORs with 95% CIs and p-values reported for all 13 candidate predictors. |
| Results | **15a** | Present the full prediction model to allow predictions for individuals. | **Yes** | Figure 2 / Table 5 | Nomogram presented with all three predictor contributions. |
| Results | **15b** | Explain how to use the prediction model. | **Yes** | Figure 2 Legend | Nomogram figure legend describes point allocation and probability estimation. |
| Results | **16** | Report performance measures (with CIs) for the prediction model. | **Yes** | Section 3 (revised) | C-index=0.695 (corrected=0.694); Brier Score=0.212; calibration slope=1.00; intercept=0.00; HL p=0.153; sensitivity/specificity table provided. |
| Results | **17** | Report results from any model-updating exercise. | **N/A** | N/A | No model updating performed. |
| Discussion | **18** | Discuss limitations of the study. | **Yes** | Section 4 — Limitations (revised) | Single-centre retrospective design; variable selection method; Level 1+2 pooling; miscarriage OR; postnatal predictors addressed. |
| Discussion | **19a** | Give an overall interpretation of the results. | **Yes** | Section 4 | Results interpreted in context of existing literature on NICU prediction and Robson classification. |
| Discussion | **19b** | Discuss the potential clinical use and implications for future research. | **Yes** | Section 4 (revised) | Clinical utility as prenatal risk stratification tool discussed; external validation identified as prerequisite. |
| Other | **20** | Provide information about supplementary resources. | **Partial** | Supplementary Material | TRIPOD+AI checklist and Supplementary Tables S1-S2 provided. Dataset available on reasonable request. |
| Other | **21** | Give the source(s) of funding and role of funders. | **Yes** | To be confirmed | Authors to confirm funding statement in final manuscript. |

*Abbreviations: C-index, concordance index; CI, confidence interval; DCA, decision curve analysis; EPV, events per variable; HL, Hosmer–Lemeshow; N/A, not applicable; NICU, neonatal intensive care unit; OR, odds ratio.*
