# Supplementary material for: Severity stratification of NICU-admitted neonates using Robson classification and obstetric risk profile: a nomogram-based study
Source: Front Med (Lausanne). 2026 Jul 8;13:1812229. doi: 10.3389/fmed.2026.1812229 (PMC13388305; doi:10.3389/fmed.2026.1812229)
Supplement: Supplementary file 2 [file Table_2.DOCX]

Supplementary Table S1. Performance of the prediction model across threshold probabilities for Level 3 NICU care

Performance metrics of the nomogram for predicting Level 3 NICU care among NICU-admitted neonates (N = 1,815; prevalence = 40.4%). PPV: positive predictive value; NPV: negative predictive value.

| Threshold Probability | TP | FP | TN | FN | Sensitivity | Specificity | PPV | NPV | Accuracy |
| --- | --- | --- | --- | --- | --- | --- | --- | --- | --- |
| 0.25 (25%) | 688 | 621 | 460 | 46 | 0.94 | 0.43 | 0.53 | 0.91 | 0.63 |
| 0.30 (30%) | 606 | 648 | 433 | 128 | 0.83 | 0.40 | 0.48 | 0.77 | 0.57 |
| 0.35 (35%) | 560 | 548 | 533 | 174 | 0.76 | 0.49 | 0.51 | 0.75 | 0.60 |
| 0.40 (40%) | **491** | **418** | **663** | **243** | **0.67** | **0.61** | **0.54** | **0.73** | **0.64** |
| 0.45 (45%) | 405 | 280 | 801 | 329 | 0.55 | 0.74 | 0.59 | 0.71 | 0.66 |
| 0.50 (50%) | 323 | 204 | 877 | 411 | 0.44 | 0.81 | 0.61 | 0.68 | 0.66 |
| 0.55 (55%) | 241 | 138 | 943 | 493 | 0.33 | 0.87 | 0.64 | 0.66 | 0.65 |
| 0.60 (60%) | 158 | 81 | 1000 | 576 | 0.22 | 0.93 | 0.66 | 0.63 | 0.64 |

*The threshold of 0.40 (40%) approximates the observed event rate (40.4%) and represents a balanced operating point between sensitivity and specificity. Abbreviations: TP, true positives; FP, false positives; TN, true negatives; FN, false negatives; PPV, positive predictive value; NPV, negative predictive value.*
